# Supplementary material for: The Effect of Surface Oxygen Coverage on the Oxygen Evolution Reaction over a CoFeNiCr High-Entropy Alloy
Source: Nanomaterials (Basel). 2024 Jun 19;14(12):1058. doi: 10.3390/nano14121058 (PMC11206725; doi:10.3390/nano14121058)
Supplement: Supplementary file 1 [file nanomaterials-14-01058-s001.zip › nanomaterials-3046854-supplementary.pdf]

Supplementary Material

The Effect of Surface Oxygen Coverage on the Oxygen Evolution Reaction over CoFeNiCr High-Entropy Alloy

Geng Yuan[1], Luis Ruiz Pestana[2,*]

[1]Chemical, Environmental and Materials Engineering, University of Miami, Coral Gables, Florida 33146, United States.

[2]Civil and Architectural Engineering, University of Miami, Coral Gables, Florida 33146, United States.

*Corresponding author: Luis Ruiz Pestana (luisruizpestana@miami.edu)

Table S1. Number of adsorption energies obtained from DFT calculations for each intermediate at different coverage ranges. We utilized varying numbers of HEA configurations and intermediate configurations based on different θ_O ranges.

Pristine surface				Oxygen-covered surface											
$\theta_O = 0$				$\theta_O \in [0.25, 0.5)$				$\theta_O \in [0.5, 0.75)$				$\theta_O \in [0.75, 1)$			
Configuration	HO*	O*	HOO*	Configuration	HO*	O*	HOO*	Configuration	HO*	O*	HOO*	Configuration	HO*	O*	HOO*
A	11	11	1	#1	5	5	3	#1	4	4	3	#1	11	10	10
				#2	5	5	4	#2	4	5	4				
				#3	5	5	2	#3	5	4	5				
				#4	5	4	3	#4	5	5	4				
				#5	5	5	3								
				#6	4	5	3								
B	10	11	1	#1	5	3	2	#1	5	5	3	#2	11	10	8
				#2	5	5	3	#2	5	5	5				
				#3	5	5	1	#3	4	5	4				
				#4	5	4	3	#4	5	5	5				
				#5	5	5	2	#5	5	5	4				
C	10	11		#1	4	5		#1	5	5	4	#3	11	11	11
				#2	5	5	2	#2	5	5	4				
				#3	5	5	2	#3	5	5	4				
				#4	5	5	3	#4	5	5	4				
				#5	5	5	4								
				#6	5	5	4								
D	8	11	1	#1	5	5	2	#1	5	5	5				
				#2	5	5	2	#2	5	5	5				
				#3	5	5	3	#3	4	5	3				
				#4	5	5	3	#4	11	11	10				
				#5	5	5	3								
				#6	4	5	2								
				#7	5	5	5								
E	8	11	2	#1	5	5	2	#1	5	5	4	#4	11	10	11
				#2	3	5	1	#2	5	5	5				
				#3	5	5	2	#3	5	5	5				
				#4	5	5	2	#4	5	5	5				
				#5	4	4	3	#5	5	5	5				
F	10	11										#5	11	9	11
G	10	11										#6	11	11	8
H	11	11	1									#7	9	11	7
I	10	11										#8	11	11	11
J	10	11										#9	11	11	11
K	10	11										#10	11	11	7
Total Number	108	121	6	Total Number	139	140	74	Total Number	112	114	100	Total Number	108	105	95

We employed a total of 11 configurations of pristine CoFeNiCr HEA, labeled alphabetically from A to K as listed in the table. These 11 pristine configurations were used to generate oxygen-covered HEA configurations across various oxygen converges. All configurations presented in this table underwent geometry optimization. Specifically, pristine configurations A, B, C, D, and E were used to generate oxygen-covered configurations with θ_O in the range [0.25,0.5) and [0.5,0.75), respectively. All 11 pristine configurations were used to generate oxygen-covered configurations with θ_O in the range [0.75,1) except for configuration D, as the coverage of oxygen-covered configuration D falls below the range [0.75,1) after optimization. In the table, the values displayed in bold, and italic represent the number of intermediate adsorption energies for each configuration. These values also represent the number of intermediate configurations for each pristine or oxygen-covered HEA configuration. The total number of adsorption energies among all oxygen coverages (including pristine configurations) is 1,222.
